# A simple phylogenetic approach to analyze hypermutated HIV proviruses reveals insights into their dynamics and persistence during antiretroviral therapy

**DOI:** 10.21203/rs.3.rs-4549934/v1

**Published:** 2024-06-13

**Authors:** Aniqa Shahid, Bradley R. Jones, Maggie C. Duncan, Signe MacLennan, Michael J. Dapp, Mark H. Kuniholm, Bradley Aouizerat, Nancie M. Archin, Stephen Gange, Igho Ofotokun, Margaret A. Fischl, Seble Kassaye, Harris Goldstein, Kathryn Anastos, Jeffrey B. Joy, Zabrina L. Brumme

**Affiliations:** Faculty of Health Sciences, Simon Fraser University, Burnaby, British Columbia, Canada; Department of Mathematics, Simon Fraser University, Burnaby, British Columbia, Canada; Faculty of Health Sciences, Simon Fraser University, Burnaby, British Columbia, Canada; Faculty of Health Sciences, Simon Fraser University, Burnaby, British Columbia, Canada; Department of Microbiology, University of Washington, School of Medicine, Seattle, Washington, USA; Department of Epidemiology and Biostatistics, University at Albany, State University of New York, Rensselaer, New York, USA; College of Dentistry, New York University, New York, New York, USA; UNC HIV Cure Center, Institute of Global Health and Infectious Diseases, University of North Carolina at Chapel Hill, North Carolina, USA; Department of Epidemiology, Johns Hopkins Bloomberg School of Public Health, Baltimore, Maryland, USA; Division of Infectious Diseases, Department of Medicine, Emory University School of Medicine, Atlanta, Georgia, USA; Division of Infectious Diseases, Department of Medicine, University of Miami School of Medicine, Miami, Florida, USA; Division of Infectious Diseases and Tropical Medicine, Georgetown University, Washington, DC, USA; Departments of Microbiology and Immunology and Pediatrics, Albert Einstein College of Medicine, Bronx, New York, USA; Department of Medicine, Albert Einstein College of Medicine, Bronx, New York, USA; Department of Medicine, University of British Columbia, Vancouver, British Columbia, Canada; Faculty of Health Sciences, Simon Fraser University, Burnaby, British Columbia, Canada

## Abstract

Hypermutated proviruses, which arise in a single HIV replication cycle when host antiviral APOBEC3 proteins introduce extensive G-to-A mutations throughout the viral genome, persist in all people living with HIV receiving antiretroviral therapy (ART). But, the within-host evolutionary origins of hypermutated sequences are incompletely understood because phylogenetic inference algorithms, which assume that mutations gradually accumulate over generations, incorrectly reconstruct their ancestor-descendant relationships. Using > 1400 longitudinal single-genome-amplified HIV *env-gp120* sequences isolated from six women over a median 18 years of follow-up − including plasma HIV RNA sequences collected over a median 9 years between seroconversion and ART initiation, and > 500 proviruses isolated over a median 9 years on ART − we evaluated three approaches for removing hypermutation from nucleotide alignments. Our goals were to 1) reconstruct accurate phylogenies that can be used for molecular dating and 2) phylogenetically infer the integration dates of hypermutated proviruses persisting during ART. Two of the tested approaches (stripping all positions containing putative APOBEC3 mutations from the alignment, or replacing individual putative APOBEC3 mutations in hypermutated sequences with the ambiguous base R) consistently normalized tree topologies, eliminated erroneous clustering of hypermutated proviruses, and brought *env*-intact and hypermutated proviruses into comparable ranges with respect to multiple tree-based metrics. Importantly, these corrected trees produced integration date estimates for *env*-intact proviruses that were highly concordant with those from benchmark trees that excluded hypermutated sequences, indicating that the corrected trees can be used for molecular dating. Use of these trees to infer the integration dates of hypermutated proviruses persisting during ART revealed that these spanned a wide age range, with the oldest ones dating to shortly after infection. This indicates that hypermutated proviruses, like other provirus types, begin to be seeded into the proviral pool immediately following infection, and can persist for decades. In two of the six participants, hypermutated proviruses differed from *env-*intact ones in terms of their age distributions, suggesting that different provirus types decay at heterogeneous rates in some hosts. These simple approaches to reconstruct hypermutated provirus’ evolutionary histories, allow insights into their *in vivo* origins and longevity, towards a more comprehensive understanding of HIV persistence during ART.

## Introduction

Antiretroviral therapy (ART) is not curative because HIV persists as an integrated provirus within a small fraction of infected cell reservoirs (Finzi et al. 1997; Finzi et al. 1999). Entry of HIV sequences into these reservoirs begins immediately following infection (Gantner et al. 2023; Whitney et al. 2014) and continues until viral suppression is achieved on ART, yielding a genetically diverse viral reservoir (Brodin et al. 2016; Brooks et al. 2020; Jones et al. 2018; Kinloch et al. 2023; Nicolas et al. 2022; Pankau et al. 2020). Only a minority (~ 2–5%) of integrated proviruses persisting on ART however are genetically intact and capable of producing replication-competent HIV; the remainder are genetically defective and cannot produce infectious virus (Bruner et al. 2016; Ho et al. 2013; Imamichi et al. 2020; Sanchez et al. 1997). Large deletions, which occur during the minus-strand synthesis step of reverse transcription, are the most common defects, followed by hypermutation (Bruner et al. 2016; Hiener et al. 2017; Ho et al. 2013; Kinloch et al. 2023; Lee et al. 2017).

Hypermutated proviruses arise in a single HIV replication cycle when host antiviral APOBEC3 proteins catalyze widespread cytidine-to-uridine deamination within the minus-strand HIV DNA genome that is produced during reverse transcription, yielding extensive guanine to adenine (G-to-A) mutations during plus-strand synthesis (Fitzgibbon et al. 1993; Goodenow et al. 1989; Vartanian et al. 1991; Vartanian et al. 1994). Hypermutation is normally deleterious, yielding stop codons in one or more HIV reading frames (Harris and Liddament 2004; Vartanian et al. 1991; Waldron 2015). As a result, hypermutated proviruses do not generally yield evolutionary descendants (Kieffer et al. 2005; Sheehy et al. 2002). Nevertheless, hypermutated sequences readily persist, typically representing 15% (though as much as > 50%) of all proviruses during long-term ART (Bruner et al. 2016; Hiener et al. 2017; Ho et al. 2013; Kinloch et al. 2023; Lee et al. 2017).

Hypermutated HIV sequences pose challenges for phylogenetic inference algorithms, which assume that mutations gradually accumulate over generations, not all at once in a single round of replication (Gorbalenya 2017). Phylogenies inferred from sequence alignments containing hypermutated proviruses will therefore inaccurately reflect the ancestor-descendant relationships of these sequences. Due to their large number of G-to-A mutations, the terminal branch lengths of hypermutated sequences are typically extended in these trees, and they will also often cluster together due to a type of phylogenetic error known as long branch attraction, whereby divergent sequences are classified as being more similar to one another simply because they have undergone a large amount of change, not because they share recent ancestry (Bergsten 2005). Though hypermutated sequences are routinely included in phylogenies simply as a way to visualize complete datasets (Halvas et al. 2020; Kearney et al. 2016; Patro et al. 2019), such trees should not be used for formal hypothesis testing. To our knowledge, no standard approaches exist to correctly infer ancestor-descendant relationships in datasets that include hypermutated sequences. Instead, these sequences are typically removed from HIV alignments, excluding them from phylogenetic hypothesis testing entirely (Bozzi et al. 2019; Brodin et al. 2016; Brooks et al. 2020; Jones et al. 2018; Jones and Joy 2023; Pinzone et al. 2019). As a result, relatively little is known about the within-host origins and longevity of hypermutated proviruses.

To address these gaps, we used longitudinal within-host HIV *env-gp120* sequence datasets from six participants of the Women’s Interagency HIV Study (WIHS) (Shahid et al. 2024) to evaluate the ability of three nucleotide alignment modification strategies to normalize the topologies of trees containing hypermutated proviruses. Using these corrected trees, we then estimated the integration dates of *env*-intact and hypermutated proviruses persisting during ART, towards better understanding the within-host evolutionary dynamics of these different proviral types.

## Methods

### Study participants and within-host HIV sequence datasets

We analyzed longitudinal, single-genome-amplified HIV *env-gp120* sequence datasets previously collected from six WIHS participants with documented HIV seroconversion (Shahid et al. 2024). WIHS is a multi-center cohort of women living with (or without) HIV in the United States (Adimora et al. 2018; Bacon et al. 2005; Barkan et al. 1998), that has now merged into the MACS/WIHS Combined Cohort Study (MWCCS) (D’Souza et al. 2021). Each participant’s longitudinal dataset comprised plasma HIV RNA *env-gp120* sequences collected between seroconversion and ART initiation, along with *env-gp120* proviral sequences sampled during ART (Shahid et al. 2024) (Table 1). All sequences were collected by single-genome amplification, where those with nucleotide mixtures, defects (*e.g.,* deletions causing frameshifts) or evidence of within-host recombination (identified using RDP4 v4.1 (Martin et al. 2015)) were excluded (Shahid et al. 2024). Sequences that were 100% identical in *env-gp120* were collapsed to a single representative sequence prior to phylogenetic inference. Within-host datasets comprised a median 242 (IQR 119–337) distinct sequences per participant.

### Ethics statement

Institutional review boards at each WIHS clinical research site approved the study protocol. All participants provided written informed consent. This nested sub-study was additionally approved by the institutional review boards at Providence Health Care/University of British Columbia, and Simon Fraser University.

### Identification of hypermutated sequences and sequence alignment modification

Hypermutated HIV sequences were identified using Hypermut 2.0, available at https://www.hiv.lanl.gov/content/sequence/HYPERMUT/hypermut.html (Rose and Korber 2000). This program takes a nucleotide alignment as input, where the first sequence is used as a reference to which all others are compared. As recommended for within-host datasets (Rose and Korber 2000), we chose the most frequently-observed *env-gp120* sequence from the first plasma HIV RNA sampling timepoint as the reference wherever possible. Hypermut defines APOBEC3 target sites as GRD; that is, a G followed by either A or G (denoted by the IUPAC code R (Cornish-Bowden 1985)), then followed by A, G or T (denoted by D), where the bold and underlined G is the APOBEC3 target site. Non-APOBEC3 target sites are defined as GY (where Y denotes C or T), or GRC. Hypermut identifies all target and non-target sites within each sequence, and categorizes each as mutated (*i.e.,* harboring an A) or not (*i.e.,* harboring a C, G or T). The program then compares the proportion of mutated target and non-target sites in each sequence using Fisher’s exact test. Sequences enriched in G-to-A mutations at target sites with p < 0.05 are identified as hypermutated.

We then prepared five within-host *env-gp120* sequence alignments for each participant, where the first two were controls and the last three used different strategies to remove hypermutation. Sequence alignments were performed in a codon-aware manner using MAFFT v7.471 (Katoh and Standley 2013) and manually inspected in AliView v1.26 (Larsson 2014). The first alignment contained all pre-ART *env-gp120* plasma HIV RNA sequences plus only the *env*-intact proviruses sampled during ART (*i.e.,* hypermutated proviruses were excluded, as is the current practice in the field (Brooks et al. 2020; Jones et al. 2018; Jones et al. 2020; Kinloch et al. 2023)). We called this the “*env*-intact only” alignment, where the resulting phylogeny was used as the benchmark for provirus molecular dating. The second alignment contained all pre-ART plasma HIV RNA sequences plus all (*i.e.,* both *env*-intact and hypermutated) proviruses sampled during ART, where the phylogeny inferred from this “HM-Unaltered” alignment served to illustrate the skewed topologies of resulting trees. The next three alignments were modifications of this second one, in which we tested different strategies to remove hypermutation and thereby normalize topology. The first strategy, HM-Stripped, removed all nucleotide positions that harbored an A at an APOBEC3 target site in at least one hypermutated sequence, yielding a shorter overall alignment. The second strategy, HM-Replacedw/R, individually replaced all A bases at APOBEC3 target sites within hypermutated sequences with R. The third strategy, HM-Replacedw/G, individually replaced all A bases at APOBEC3 target sites within hypermutated sequences with G. Both these strategies preserved the alignment length. Here, replacing with G assumes that all A bases at target sites are the result of APOBEC3 effects, whereas replacing with R recognizes the possibility that some may be legitimate A bases that are not attributable to APOBEC3 effects. Visualizations of the HM Unaltered, HM-Stripped and HM-Replacedw/R alignments are provided in **Supplementary Fig. 1**. Phylogenies inferred from these alignments were evaluated as described below.

### Within-host phylogenetic inference, rooting and tree metrics

Maximum likelihood phylogenies were inferred from sequence alignments following automated model selection using an Akaike information criterion (AIC) in IQ-TREE 2. Best-fit models are reported in **Supplementary Table 1**. Branch support values were derived using the ultrafast bootstrap option (1,000 bootstraps) (Hoang et al. 2018; Minh et al. 2020). Phylogenies were visualized using the R package *ggtree* (Yu 2020).

Most of our downstream analyses required rooting the tree at the inferred most recent common ancestor (MRCA) of the dataset. As previously described, we used a modified root-to-tip regression approach where we explored all positions in the tree to identify the location that maximized the (Pearson’s) correlation between the root-to-tip distances of *all plasma HIV RNA sequences collected prior to ART initiation,* and their sampling dates (Jones et al. 2018). This location was set as the tree root, which represents the estimated transmitted/founder virus, or a close descendant thereof, in these datasets.

**Figure 1:**
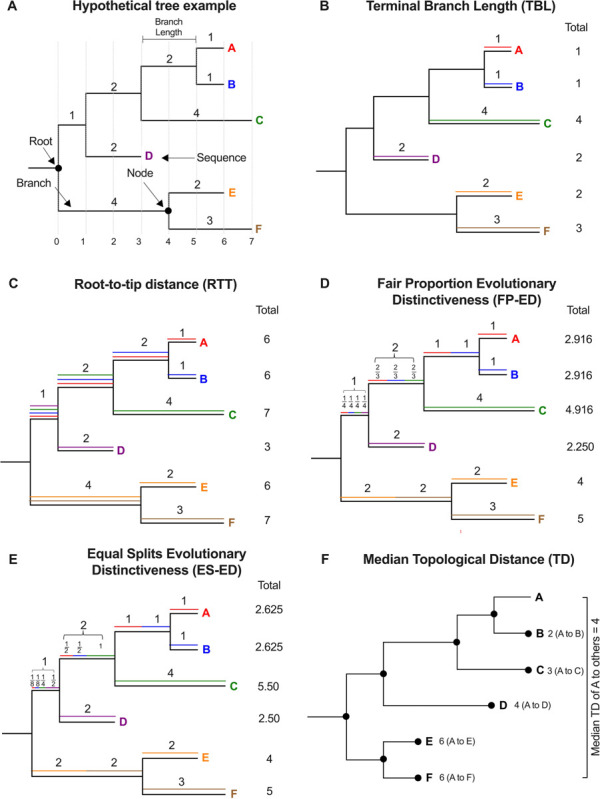
Tree-based metrics (A) Hypothetical tree containing six sequences, labeled A through F, each in a unique color. Vertical dotted lines depict the distance scale in hypothetical units numbered below the tree. All other panels depict this same tree. (B) Colored horizontal lines trace the terminal branch lengths (TBL) of sequences A-F, with the values also shown at the right of the tree. (C) Each sequence’s path from root to tip is traced with a unique color, where the sum of these lengths (representing the root-top-tip distance; RTT) is shown at the right of the tree. (D and E) *Fair Proportion Evolutionary Distinctiveness* (FP-ED) divides the shared evolutionary history represented by an internal branch equally among all its descendant sequences at the tips. Here, colored lines and associated fractional branch lengths show how internal branch lengths are apportioned to each sequence. The sum of each sequence’s branch measurements, the FP-ED, is shown at the right of the tree. (E) In contrast, *Equal Splits Evolutionary Distinctiveness* (ES-ED) assigns 50% of each internal branch length to each immediate descendant. As such, branches leading to a single descendant assign 50% of that branch to this descendant, whereas branches leading to multiple descendants further split the remaining 50% among them using this same scheme. The sum of these measurements, the ES-ED, is shown at the right of the tree. (F) The topological distance (TD) separating sequence A from all others is shown to the right of each tip, where TD is computed as the total number of nodes separating A from all others in the tree. Here, the median TD separating A from all others in the tree is 4.

To evaluate the extent to which the three alignment modification strategies normalized the position of hypermutated proviruses in the tree, we compared *env*-intact and hypermutated proviruses with respect to various tree-based metrics, explained in Fig. 1. We quantified *terminal branch length* (TBL), which is the length of the branch connecting each sequence to the tree, in estimated substitutions per nucleotide site (Fig. 1B). We computed *root-to-tip distance (RTT*), which is the total distance between each tip and the tree root (Fig. 1C). We computed two measures of evolutionary distinctiveness: *Fair Proportion Evolutionary Distinctiveness* (FP-ED) and *Equal Splits Evolutionary Distinctiveness* (ES-ED), both of which distribute the root-to-tip distances in a tree among the descendant sequences at the tips (Pavoine 2017). FP-ED does this by dividing the shared evolutionary history represented by an internal branch equally among all its descendant tips, regardless of branching order (Isaac et al. 2007; Redding et al. 2014) (Fig. 1D), whereas ES-ED assigns a longer portion of shared internal branches to immediate descendants (Redding and Mooers 2006) (Fig. 1E). FP-ED and ES-ED were computed using a custom R script with package *picante* (v1.8.2) (Kembel et al. 2010). We computed each proviral sequence’s median *topological distance (TD)* from all other sequences of the same type (*i.e., env*-intact or hypermutated), where distance was defined as the *number of nodes* separating each pair (Fig. 1F). Finally, we used the Slatkin-Maddison (SM) test (Slatkin and Maddison 1989), implemented using the R package *slatkin.maddison* (v0.1.0; https://github.com/prmac/slatkin.maddison) to assess the extent to which *env*-intact and hypermutated sequences displayed population structure in the tree. This test determines the minimum number of migrations between groups to explain the distribution of groups at the tree tips: the smaller the number, the stronger the support for population structure. Statistical support is based on the number of migrations that would be expected in a randomly-structured population, simulated by permuting group labels between tips. Note that Slatkin-Maddison returns an estimated p-value, where a value of 0 can be interpreted as p < 0.001, as 1,000 permutations were performed.

### Within-host phylogenetic inference and proviral dating

We inferred the integration dates of *env*-intact and hypermutated proviruses persisting during ART using a published phylogenetic approach (Jones et al. 2018). Using the rooted trees, we fit a linear model relating the root-to-tip distances of *pre-ART plasma HIV sequences* to their collection dates. The slope of this line represents the average within-host *env-gp120* evolutionary rate during untreated HIV infection, and the x-intercept represents the inferred root date. Model quality was assessed by comparing the model’s AIC to that of a null model with zero slope. To pass quality control (QC), the linear model needed to have an AIC value at least 10 units lower than the null model (ΔAIC ≥ 10), and a root date prior to the first plasma sampling. All phylogenies met these criteria (**Supplementary Table 1**). We then used the linear model to convert proviral root-to-tip distances to their integration dates. The custom R script for this method is available at https://github.com/cfe-lab/phylodating.

### Statistical analysis

Spearman’s correlation (ρ) and Lin’s concordance correlation coefficient (ρc) were calculated in R. All other statistical analyses were performed in Prism, v10.0.2 (GraphPad Software). A threshold of p < 0.05 was used to denote statistical significance.

## Results

### Within-host HIV sequence datasets

We analyzed 1,408 single-genome-amplified HIV *env-gp120* sequences collected longitudinally from six WIHS participants who experienced HIV seroconversion (a seventh participant from the original study was not included here, as no hypermutated proviruses were isolated from their samples) (Shahid et al. 2024) (Table 1). The data included 866 distinct HIV RNA *env-gp120* sequences (median 157 per participant) isolated from plasma over a median of 9 time points spanning a median of 7 years between seroconversion and ART initiation. The data also included 542 distinct *env-gp120* proviral sequences, including 449 *env*-intact ones (median 62 per participant) and 93 hypermutated ones (median 19 per participant) isolated from peripheral blood at a minimum of 3 time points over a median of 8.7 years during ART (Table 1). All participants had HIV subtype B, with no evidence of dual or super-infection.

### Identifying sites of hypermutation

Between 7 and 42% of participants’ proviral sequences were hypermutated (though hypermutation was not observed in plasma HIV RNA sequences, as expected). In a given within-host alignment, between 9–11% of *env-gp120* nucleotide positions had a putative APOBEC3-driven A in at least one sequence (Table 2). Hypermutated proviruses harbored a grand median of 45 putative APOBEC3 mutations (representing 31% of all possible target sites, and 3% of all *env-gp120* nucleotides), but the overall range was 9 to 83 putative APOBEC3 mutations per *env-gp120* sequence (representing 6–61% of all possible target sites, and 0.6–5% of all *env-gp120* nucleotides). For context, the grand median of putative APOBEC3 mutations in *env*-intact (non-hypermutated) proviruses was 5.

### Assessing how alignment modification strategies normalized tree topology and metrics

We next investigated how well our sequence alignment modification strategies helped normalize tree topologies, beginning with participant WIHS-P2 as an example.

**Figure 2:**
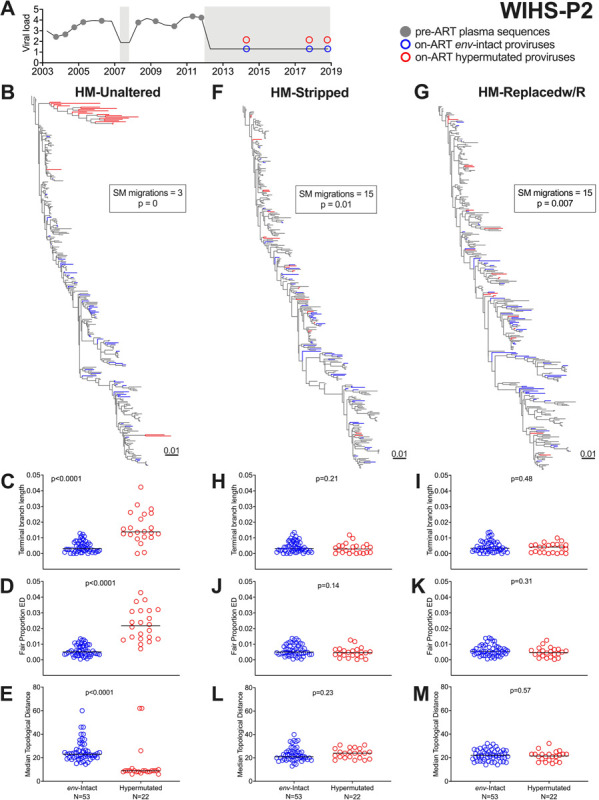
WIHS-P2: clinical history, within-host phylogenies and tree metrics. (A) Participant WIHS-P2’s plasma viral load history and sampling timeline. Closed grey circles denote pre-ART plasma HIV RNA sampling. Open circles denote proviral sampling on ART (blue for *env*-intact proviruses and red for hypermutated proviruses). Grey shading denotes ART. (B) Participant WIHS-P2’s rooted maximum-likelihood phylogeny, inferred from all within-host *env-gp120* sequences including hypermutated proviruses. Branches are colored by sequence type (pre-ART HIV RNA = grey; on-ART *env*-intact provirus = blue; on-ART hypermutated provirus = red). Inset shows the number of inferred migrations between *env*-intact and hypermutated sequence groups computed using the Slatkin-Maddison (SM) test, along with the estimated p-value. Here, p = 0 can be interpreted as p < 0.001, as 1,000 permutations were performed. (C) Terminal Branch Lengths (TBL) of *env*-intact and hypermutated sequences in this tree. Horizontal black lines denote the median values. P-value computed using the Mann-Whitney U-test. (D) Fair Proportion Evolutionary Distinctiveness (FP-ED) values for *env*-intact and hypermutated sequences in this tree. (E) Median Topological distances (TD) separating *env*-intact and hypermutated proviruses from others of the same type (F-L) same as panels B through E, but for the phylogeny inferred from an alignment where positions containing hypermutation were stripped out. (G-M) same as panels B through E, but for the phylogeny inferred from an alignment where hypermutated sites were replaced with R.

Participant WIHS-P2’s dataset included 227 plasma HIV RNA *env-gp120* sequences sampled over 9 years during untreated infection, and 75 proviruses (53 *env*-intact, 22 hypermutated) sampled over ~7 years during ART (Fig. 2A). WIHS-P2’s unmodified nucleotide alignment yielded a phylogeny that placed nearly all hypermutated proviruses into a single clade with high (≥ 90%) bootstrap support (Fig. 2B; a larger tree with branch support values is shown in **Supplementary Fig. 2**). Hypermutated provirus terminal branch lengths in this tree were on average four times longer than *env*-intact ones (p < 0.0001; Fig. 2C), though their root-to-tip distances were not significantly inflated (p = 0.2, **Supplementary Fig. 3A**). Hypermutated proviruses also exhibited significantly higher evolutionary distinctiveness (ED) than *env*-intact ones in this tree (p < 0.0001 for both fair proportion and equal splits ED; Fig. 2D and **Supplementary Fig. 4A**). Also reflecting the erroneous clustering of hypermutated sequences in this tree, the median number of nodes separating hypermutated sequences from one another (*i.e.,* topological distance) was on average only half of that separating *env*-intact proviruses (p < 0.0001; Fig. 2E). A Slatkin-Maddison test also returned significant evidence of genetic population structure (*i.e.,* “compartmentalization”) between hypermutated and *env*-intact proviruses in this tree (three inferred migrations; estimated p = 0; Fig. 2B **inset**).

By contrast, the tree inferred from WIHS-P2’s HM-Stripped alignment, in which 140 (of 1515) *env-gp120* positions harboring putative APOBEC3 mutations had been removed, exhibited a substantially normalized topology (Fig. 2F). The same was true for the tree inferred from the HM-Replacedw/R alignment, where a median of 43 putative APOBEC3-driven A bases in hypermutated sequences had been replaced with R (Fig. 2G; larger trees in **Supplementary Fig. 2**). In both trees, hypermutated proviruses were now comparable to *env*-intact ones in terms of terminal branch lengths (both p > 0.1; Figs. 2H and 2I), evolutionary distinctiveness (all p > 0.1; Figs. 2J and 2K; **Supplementary Figs. 4B and 4C**) and topological distance (both p > 0.1, Figs. 2L and 2M). Genetic compartmentalization between *env*-intact and hypermutated proviruses was also markedly reduced (15 inferred migrations compared to the original 3), though the p-values remained marginally significant (both p ≤ 0.01; Figs. 2F and 2G, **insets**). Of note, root-to-tip distances of hypermutated proviruses in these two trees were now shorter than those of *env*-intact ones (both p < 0.001; **Supplementary Figs. 3B and 3C**). In contrast, while the tree inferred from participant WIHS-P2’s HM-Replacedw/G alignment (where putative APOBEC3-driven A bases in hypermutated sequences were replaced with G) appeared broadly normalized, *env*-intact and hypermutated sequences remained highly significantly compartmentalized in this tree (estimated p = 0; **Supplementary Fig. 5**).

**Figure 3:**
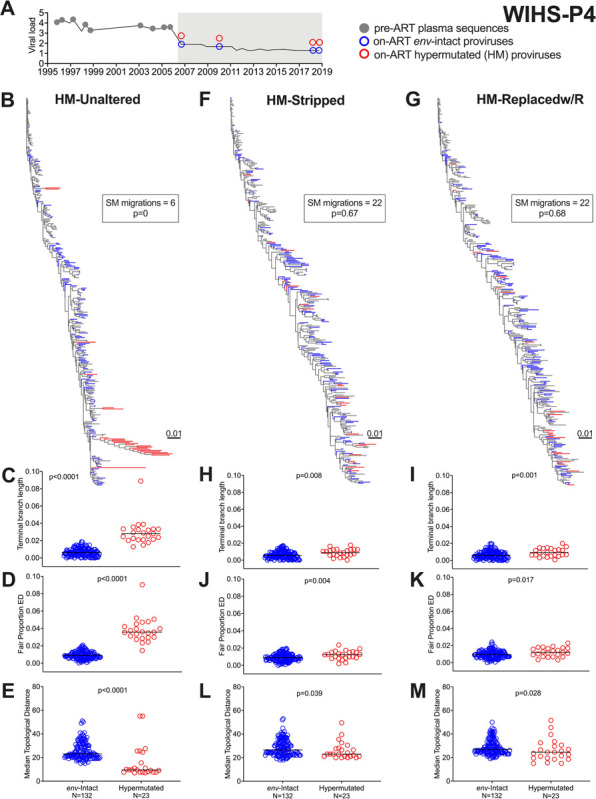
WIHS-P4 clinical history, within-host phylogenies and tree metrics. Legend as in Figure 2, except the data are for WIHS-P4.

As our second example, participant WIHS-P4’s dataset included 182 plasma HIV RNA *env-gp120* sequences sampled over ~ 11 years pre-ART, and 155 proviruses (132 *env*-intact; 23 hypermutated) sampled during 12 years of ART (Fig. 3A). The unaltered alignment produced a phylogeny (Fig. 3B; larger tree in **Supplementary Fig. 6**) where hypermutated sequences exhibited significantly inflated branch lengths, root-to-tip distances and evolutionary distinctiveness (all p < 0.0001; Figs. 3C and 3D, **Supplementary Figs. 3D and 4D**), erroneous clustering (p < 0.0001 Fig. 3E) and significant compartmentalization (estimated p = 0; Fig. 3B **inset**). By contrast, the HM-Stripped and HM-Replacedw/R alignments produced substantially normalized trees (Figs. 3F and 3G respectively; larger trees in **Supplementary Fig. 6**) with no genetic compartmentalization between *env*-intact and hypermutated sequences (22 migrations compared to the original 6; both p > 0.1; Figs. 3F and 3G **insets**). The ranges of terminal branch lengths, root-to-tip distance measurements, evolutionary distinctiveness measures and topological distances were now also comparable between *env*-intact and hypermutated proviruses, though the latter remained modestly yet statistically significantly different from *env*-intact sequences by most measures (p-values from 0.001 to 0.039, Figs. 3H to 3M; **Supplementary Figs. 3E and 3F; Supplementary Figs. 4E and 4F**). In contrast, hypermutated sequences remained highly compartmentalized in the phylogeny inferred from WIHS-P4’s HM-Replacedw/G alignment (**Supplementary Fig. 7**).

**Figure 4:**
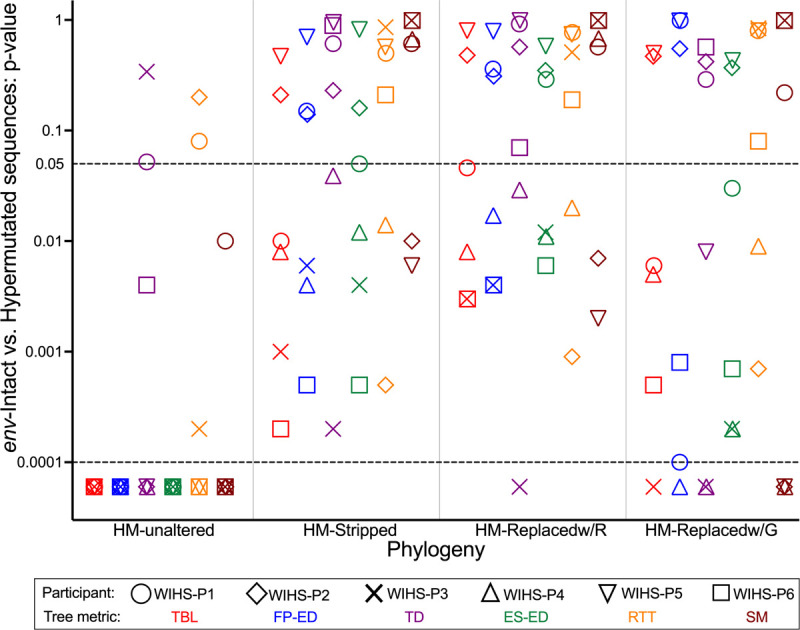
Summary of tree metrics across all participants. For each participant (each shown with a distinct symbol), the p-value derived from comparing *env*-intact and hypermutated proviruses in the tree for each of the phylogenetic metrics (each shown in a distinct color) is plotted for each tree type. TBL = Terminal Branch Length; FP-ED = Fair Proportion Evolutionary Distinctiveness; TD = Topological Distance; ES-ED = Equal Splits Evolutionary Distinctiveness; RTT = root-to-tip distance; SM = Slatkin-Maddison. For consistency with the other metrics, SM estimated p-values of 0 are shown here as p < 0.0001. The horizontal dashed line at p = 0.05 denotes the standard threshold for statistical significance.

The same analyses were applied to participants WIHS-P1, WIHS-P3, WIHS-P5, and WIHS-P6 (small trees and select metrics in **Supplementary Figs. 8–11**; large trees in **Supplementary Figs. 12–15;** remaining metrics in **Supplementary Figs. 3 and 4**). Broadly, the trees inferred from the HM-Stripped and HM-Replacedw/R alignments were markedly normalized and yielded metric values for *env*-intact and hypermutated proviruses that spanned comparable ranges. For some participants, these metrics normalized such that *env*-intact and hypermutated viruses became statistically comparable (*e.g.,* WIHS-P5; **Supplementary Fig. 10)**. For others, hypermutated sequences remained somewhat distinctive (*e.g.,* hypermutated provirus terminal branch lengths and evolutionary distinctiveness remained slightly elevated for WIHS-P6; **Supplementary Figs. 4 and 11**), but in all cases these differences were far smaller in magnitude than those from the trees inferred from unaltered alignments. Indeed, the p-values derived from comparing *env*-intact and hypermutated proviruses in the HM-Stripped and HM-Replacedw/R trees were an average > 3 logs higher than those from the HM-Unaltered trees, with 56% of comparisons yielding p-values > 0.05 (Fig. 4).

By contrast, the HM-Replacedw/G approach did not reliably normalize the trees. In particular, WIHS-P5’s HM-Replacedw/G phylogeny maintained obvious clustering of hypermutated sequences and very strong compartmentalization, while terminal branch lengths, fair proportion evolutionary distinctiveness, and topological distance also remained highly skewed for one or more participants (Fig. 4, and data not shown). As such, only the HM-Stripped and HM-Replacedw/R trees were advanced to further evaluation.

### Inferring proviral integration dates from corrected trees: a validation

**Figure 5:**
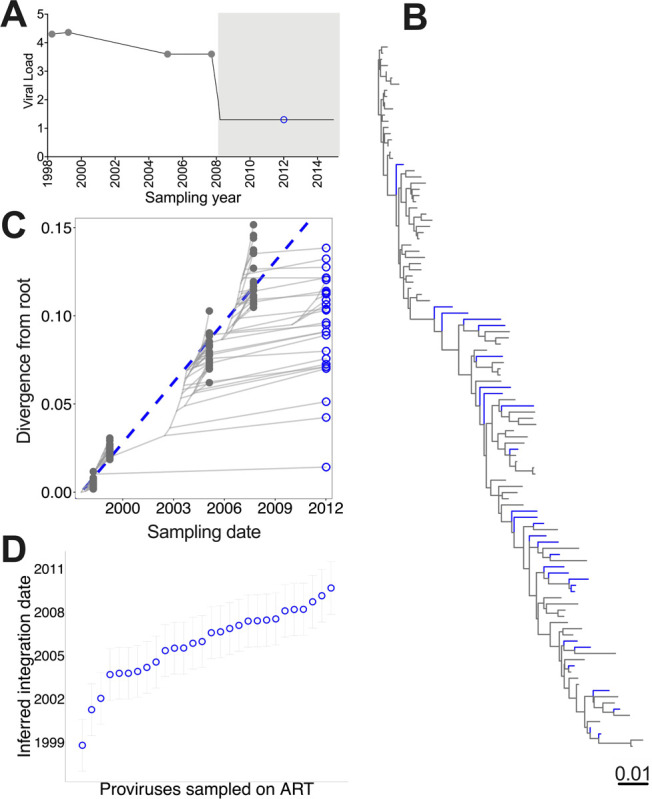
Within-host phylogenetic approach to infer proviral integration dates. (A) Viral load and sampling timeline for a hypothetical participant. Closed grey circles denote plasma HIV RNA sampling prior to ART, while the open blue circle denotes proviral HIV DNA sampling during ART. Light grey shading represents ART. (B) Rooted, maximum likelihood within-host phylogeny, with branches colored by sequence type (grey = pre-ART plasma HIV RNA; blue = on-ART proviruses). (C) HIV sequence divergence from the root over time. The blue dashed diagonal represents the linear model relating the root-to-tip distances of distinct pre-ART plasma HIV RNA sequences (closed grey circles) to their sampling dates. This model is used to convert the root-to-tip distances of proviral sequences sampled during ART (open blue circles) to their integration dates. Faint grey lines trace the ancestral relationships between HIV sequences. (D) Integration date point estimates (and 95% confidence intervals) for each distinct provirus sequence sampled during ART, sorted from oldest to youngest. The provirus shown at the bottom right of panel C for example, was estimated to have integrated in October 1998, and is shown at the bottom left of panel D.

We next investigated whether accurate evolutionary information can be extracted from these corrected trees, by phylogenetically inferring the integration dates of proviruses sampled during ART. Figure 5 illustrates how this is done. Brie y, we first root the phylogeny at the location that maximizes the correlation between the root-to-tip distances of the *pre-ART plasma HIV RNA sequences* and their sampling dates (proviruses sampled during ART, though included in the tree, are *not* considered in this correlation; Fig. 5B). This root represents the MRCA of the dataset (*i.e.,* the estimated the founder virus). We then fit a linear model relating the root-to-tip genetic distances of the pre-ART plasma sequences to their sampling dates (Fig. 5C). This model is then used to convert the root-to-tip distance of each on-ART provirus to its inferred integration date (plus 95% confidence interval; Fig. 5D).

**Figure 6:**
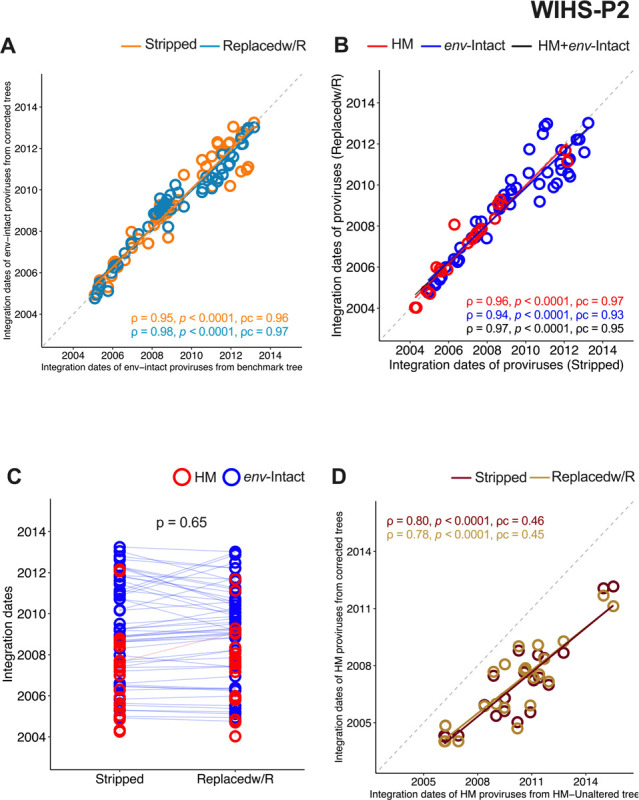
Inferring proviral integration dates from corrected trees: validation using WIHS-P2’s data. (**A**) Correlation between inferred integration dates of *env*-intact proviruses from the benchmark versus corrected trees, where the dates inferred from the HM-Stripped tree are in orange and those inferred from the HM-Replacedw/R tree are in teal. Spearman’s ρ, associated p-value, and Lin’s concordance correlation coefficient (ρc) are shown for each comparison. Regression lines in matching colors are also provided to help visualize these relationships. The dotted diagonal denotes a hypothetical perfect concordance. (B) Correlation between inferred integration dates of *all* proviruses from HM-Stripped versus HM-Replacedw/R trees, with hypermutated proviruses in red and *env*-intact proviruses in blue. Statistics are computed for all proviruses (black), hypermutated proviruses only (red) and *env*-intact proviruses only (blue). (C) Inferred integration dates of *env*-intact and hypermutated proviruses from HM-Stripped and HM-Replacedw/R trees, presented as paired measurements connected with matching-colored lines. P-value computed using the Wilcoxon matched-pairs signed rank test. (D) Correlation between inferred integration dates of hypermutated proviruses from the HM-Unaltered and corrected trees (HM-Stripped tree = maroon; HM-Replacedw/R tree = gold).

Application of this approach to WIHS-P2’s unaltered and corrected trees yielded estimated root dates that were consistent with the clinically-estimated infection date (Table 1) and comparable to the root date inferred from the benchmark (*env*-intact only) tree (**Supplementary Table 1;** the likely reason that the unaltered tree produced reasonable root dates and evolutionary rate estimates is because these metrics are computed from pre-ART plasma HIV RNA sequences only). We next verified the extent to which the integration dates of *env*-intact proviruses inferred from the corrected trees matched those inferred from the benchmark tree (which, as per current field standards, excluded hypermutated sequences entirely). Reassuringly, *env*-intact proviral integration dates inferred from the HM-Stripped tree were highly concordant with those inferred from the benchmark tree (Spearman’s rho [ρ] = 0.95, p < 0.0001; Lin’s concordance correlation coefficient [ρc = 0.96], as were those inferred from the HM-replacedw/R tree (ρ = 0.98, p < 0.0001; ρc = 0.97) (Fig. 6A). These results indicate that WIHS-P2’s corrected trees can be used for molecular dating, and produce valid proviral integration dates.

We next inferred the integration dates of all proviruses from the corrected trees, including the hypermutated ones. Inferred integration dates were highly concordant between the two approaches, yielding ρc between 0.93 and 0.97 depending on whether we compared *env*-intact, hypermutated or all proviruses (Fig. 6B). Moreover, there was no bias between the two methods (p = 0.65) (Fig. 6C). Thus, for participant WIHS-P2, both methods recovered proviral ages equally well. By contrast, the phylogeny inferred from the unaltered alignment produced hypermutated provirus integration dates that were poorly concordant with those from the corrected trees (HM-Stripped ρc = 0.46; HM-Replacedw/R ρc = 0.45; Fig. 6D). This illustrates the pitfalls of inferring evolutionary information from the former tree type.

**Figure 7:**
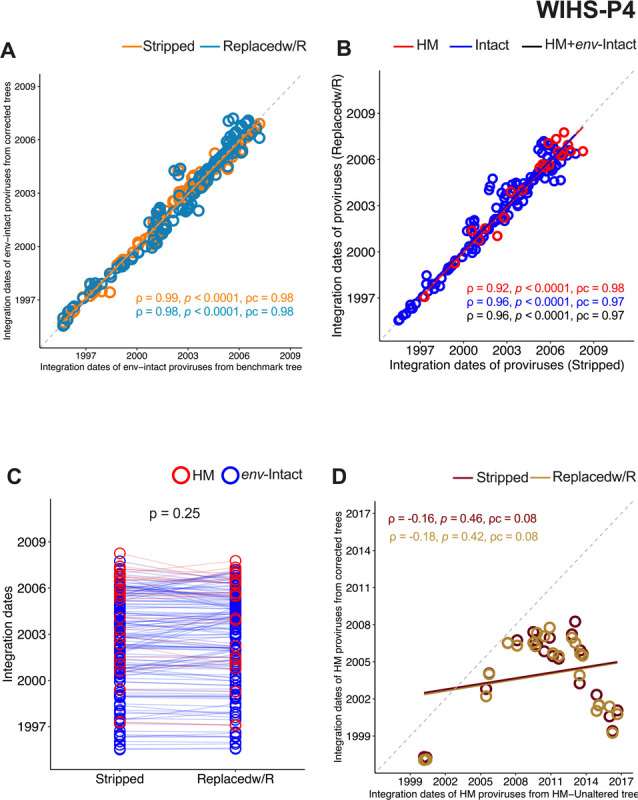
Inferring proviral integration dates from corrected trees: validation using WIHS-P2’s data. Legend as in Figure 6, except for WIHS-P4.

We obtained similar results for WIHS-P4. Again, the integration dates of *env*-intact proviruses inferred from both corrected trees were highly concordant with those inferred from the benchmark tree (both ρc = 0.98; Fig. 7A), indicating that the corrected trees are appropriate for molecular dating. Moreover, proviral integration dates inferred from the corrected trees were highly concordant with one another (ρc 0.97 to 0.98) (Fig. 7B), and showed no bias between methods (p = 0.25) (Fig. 7C). By contrast, the phylogeny inferred from the unaltered alignment produced hypermutated provirus integration dates that were highly discordant with those inferred from the corrected trees (both ρc = 0.08; Fig. 7D), again illustrating the pitfalls of inferring evolutionary information from the former tree type.

WIHS-P1, WIHS-P3, WIHS-P5, and WIHS-P6’s corrected trees similarly produced *env*-intact proviral integration dates that were strongly concordant with those inferred from their benchmark trees (ρc: 0.81 to 0.93), and generally highly concordant proviral integration dates to one another, with no bias between methods (**Supplementary Figs. 16–19**). Again, the phylogenies inferred from their unaltered alignments produced hypermutated provirus integration dates that were generally poorly concordant with those inferred from the corrected trees.

Together, these observations demonstrate that removing hypermutation from alignments is possible, and yields phylogenies that can be used to infer the integration dates of both hypermutated and *env*-intact proviruses.

### Longevity and dynamics of hypermutated proviruses persisting on ART

**Figure 8:**
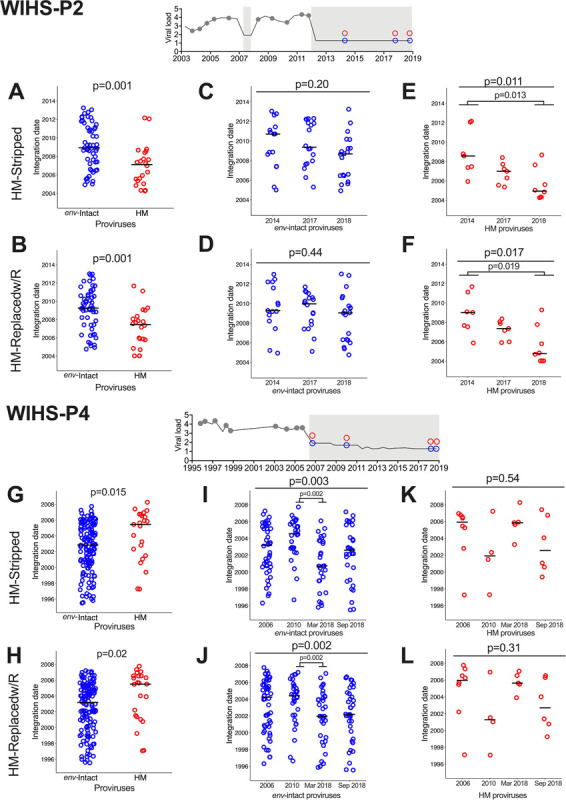
WIHS-P2 and P4: Integration dates of env-intact and hypermutated proviruses persisting during ART. Top: HIV plasma viral load and sampling history for participant WIHS-P2. (A, B) Integration dates of *env*-intact (blue) and hypermutated proviruses (HM; red) inferred from the HM-Stripped (panel A) and HM-Replacedw/R (panel B) trees. All proviruses of the same type are grouped together regardless of sampling date on ART. P-value derived from the Mann-Whitney U-test. Horizontal black lines represent the median values. (C-D) These are the same *env*-intact provirus integration dates as shown in panels A and B, but now stratified by sampling date on ART. P-value is from a Kruskal-Wallis test comparing all groups. (E-F) These are the same hypermutated provirus integration dates as shown in panels A and B, but now stratified by sampling date on ART. The large P-value at the top is from a Kruskal-Wallis test comparing all groups. The smaller p-values below represent the significant pairwise post-tests after correction for multiple comparisons. (G-L) Same as for panels A-F, except for participant WIHS-P4.

Having demonstrated that proviral integration dates can be inferred from the corrected trees, we compared the integration dates of *env*-intact and hypermutated proviruses persisting on ART. Again, we begin with participant WIHS-P2. Both of this participant’s corrected trees indicated that the hypermutated proviruses, like the *env*-intact ones, spanned essentially the entire duration of untreated infection, with the earliest dating to early 2004, approximately one year after seroconversion, (Figs. 8A and 8B). On average however, hypermutated proviruses were older than *env*-intact ones in this participant (both trees p = 0.001; Figs. 8A and 8B). Longitudinal analysis further revealed that, while integration date distributions of *env*-intact proviruses remained stable during the first seven years of ART (both trees p ≥ 0.1; Figs. 8C and 8D), hypermutated proviruses gradually shifted towards earlier integration dates over time (both trees p < 0.02; Figs. 8E and 8F), presumably because those with more recent integration dates were preferentially eliminated during long-term ART.

WIHS-P4’s proviruses also spanned essentially the entire duration of untreated infection (Figs. 8G and 8H). In contrast to WIHS-P2 however, the integration dates of their hypermutated proviruses were on average more recent than their *env*-intact ones (both trees p ≤ 0.02; Figs. 8G and 8H). As previously reported (Shahid et al. 2024), WIHS P4’s *env*-intact proviruses gradually shifted towards earlier integration dates over time on ART (both trees p ≤ 0.003; Figs. 8I and 8J), likely because those with more recent integration dates decayed more rapidly following ART initiation. In contrast, hypermutated provirus integration date distributions remained stable during ART (both trees p > 0.1; Figs. 8K and 8L).

WIHS-P1, WIHS-P3, WIHS-P5, and WIHS-P6’s hypermutated proviruses also spanned broad age ranges, but in contrast to WIHS-P2 and WIHS-P4, they did not differ from *env*-intact ones in terms of their overall integration date distributions (**Supplementary Figs. 20 and 21**). As reported previously, their *env*-intact proviral integration date distributions remained stable except for participant WIHS-P5 in whom the proviral pool shifted slightly towards later integration dates over time (**Supplementary Figs. 21C and 21D**) (Shahid et al. 2024). Hypermutated proviral integration date distributions were also stable over time except in WIHS-P1, whose proviral date distributions differed markedly by visit (**Supplementary Figs. 20E and 20F**). Though this could suggest dynamic changes over time, limited sampling must be acknowledged. Notably, the HM-Stripped and HM-Replacedw/R approaches produced comparable results except in the temporal analysis of *env*-intact proviruses for WIHS-P3, where HM-Stripped suggested a modest shift towards more recent integration dates over time, whereas HM-Replacedw/R indicated no change (**Supplemental Figs. 20I and 20J**).

## Discussion

Though hypermutated proviruses persist in all people living with HIV (PLWH) (Bruner et al. 2016; Ho et al. 2013; Kinloch et al. 2023), we know relatively little about their within-host origins because they cannot be readily incorporated into phylogenies. We explored three simple approaches to remove hypermutation from nucleotide alignments, with the dual goals of 1) reconstructing phylogenies that accurately reconstruct the within-host evolutionary histories of hypermutated sequences and 2) applying molecular dating approaches to these trees to gain insights into the within-host origins and longevity of hypermutated proviruses.

Of the approaches we evaluated, stripping nucleotide positions containing putative APOBEC3 mutations from the alignment, or replacing individual APOBEC3 mutations in hypermutated sequences with R, consistently normalized tree topologies and metrics. By contrast, replacing APOBEC3 mutations in hypermutated sequences with G failed to consistently resolve their erroneous clustering in the tree. We speculate that this is because G replacement is an overcorrection, as not all A bases at target sites are necessarily the result of APOBEC3 activities (the HIV genome is naturally high in A bases (Kypr and Mrazek 1987; Kypr et al. 1989)). Across-the-board G replacement therefore likely obscures some legitimate ancestral information (*i.e.,* inherited A bases), leaving these sequences at continued risk of long-branch attraction. By contrast, replacing putative APOBEC3 mutations with R mitigates this risk by acknowledging this ambiguity. We therefore advise against replacement of APOBEC3 mutations in hypermutated sequences with G.

We further showed that the integration dates of *env*-intact proviruses inferred from the HM-Stripped and HM-Replacedw/R approaches were highly concordant with those inferred from benchmark trees that excluded hypermutated sequences entirely, as is the current practice. The demonstration that these corrected trees provide valid molecular dating results is important because it provides, for the first time, an approach to study the within-host evolutionary origins and longevity of the large and genetically diverse population of hypermutated proviruses that persist in all PLWH during ART.

Proviral integration date estimates produced by the two approaches were highly concordant, and there was no clear difference in their performance. While the p-values derived from comparing the tree-based metrics of *env*-intact and hypermutated sequences, shown in Fig. 4, are overall slightly higher for the HM-Replacedw/R compared to the HM-Stripped approach, we caution against interpreting this to mean that the former is superior. Though we applied statistical tests to guide interpretation, the main goal was to produce tree metric values for hypermutated and *env*-intact sequences that were in the same range as one another. Both HM-Stripped and HM-Replacedw/R approaches achieved this. We did not necessarily expect that *env*-intact and hypermutated sequence metrics would all normalize completely (*i.e.,* produce non-significant p-values) because some evolutionary attributes of *env*-intact and hypermutated sequences might plausibly differ. As hypermutated sequences don’t normally yield descendants for example, their closest neighbors in the tree might be more distant than those for *env*-intact proviruses, simply because of the lower likelihood of sampling a close relative (which, for a hypermutated sequence, could only be an ancestor). Differential evolutionary dynamics between hypermutated and *env*-intact proviruses could also produce differential root-to-tip measurements (and by extension integration date estimates) between groups, a phenomenon that was indeed observed in WIHS-P2 and WIHS-P4.

We therefore offer the following considerations when choosing an approach. Since the HM-Replacedw/R approach retains the full alignment, it should also preserve more phylogenetic signal than the HM-Stripped approach, where an average of 9% of each *env-gp120* alignment was removed. This could be advantageous for HIV regions that are relatively conserved, yet hotspots for APOBEC3 mutation, for example parts of pol (Kieffer et al. 2005; Kijak et al. 2008). But, before implementing the Replacedw/R approach, it is essential to verify that the chosen phylogenetic inference package supports ambiguous characters. IQ-TREE 2, used in the present study, assigns equal likelihood to each component character (Minh et al. 2020), but other packages, such as the approximate maximum likelihood algorithm FastTree, treat all non-ACTG characters as missing data (Price et al. 2010).

It is also important to recognize when sequence alignment modifications are warranted. For routine phylogenetic visualization of HIV datasets, hypermutated sequences can be incorporated directly. Such trees might even be adequate for some limited tree-based inferences, as suggested by our finding that uncorrected trees produced reasonable root dates and evolutionary rates, likely because these calculations only use information from pre-ART plasma HIV RNA sequences. Nevertheless, our demonstration that uncorrected trees erroneously reconstructed the ancestry of hypermutated proviruses, and produced inaccurate (and often nonsensical) integration dates for them underscores why they can’t be used to answer questions about the evolutionary history of hypermutated proviruses. For such questions, the above alignment modification approaches should be used.

Our results also reveal insights into hypermutated provirus evolutionary dynamics. Like *env*-intact ones, hypermutated proviruses spanned a broad age range. From WIHS-P2 for example, we isolated hypermutated proviruses that had integrated as early as a year following seroconversion. This indicates that hypermutated proviruses, like other provirus types, begin to be seeded into the proviral pool essentially immediately following transmission, and can persist for decades thereafter. Our results also revealed evidence of differential evolutionary dynamics of hypermutated and *env*-intact proviruses in two of the six participants studied, namely WIHS-P2, whose hypermutated proviruses were on average older than *env*-intact ones, and WIHS-P4, in whom the opposite was observed. This suggests that the decay rates of different types of proviruses can be heterogeneous within a given host, as well as heterogeneous between hosts.

Our study has some limitations. We analyzed the present dataset (Shahid et al. 2024) because it is among the most comprehensive of its type (in terms of sequence N, follow-up time and sampling near seroconversion) and because *env-gp120* is commonly used for within-host HIV evolutionary studies (Brooks et al. 2020; Dapp et al. 2017). That said, participants WIHS-P3 and WIHS-P6 had only modest numbers of hypermutated proviruses, which limited our power to detect differences between these and *env*-intact proviruses in their data. Furthermore, while our proposed method should be applicable to any HIV gene region, we did not explicitly investigate this. The identification of hypermutated sequences, on which our method depends, is by definition imperfect, as it relies on a statistical cut-off and can be subtly influenced by the choice of reference sequence, particularly if a heterologous sequence (e.g. HXB2 HIV reference strain) is used for this purpose (Rose and Korber 2000). As recommended, we used the most frequent sequence observed post-seroconversion as the reference (Rose and Korber 2000), though we verified that use of a different sequence impacted the identification of hypermutated sequences minimally or not at all (*e.g.,* using an arbitrarily-chosen reference sequence from WIHS-P2’ earliest sampling time point yielded 137 (out of 1515) nucleotide positions with putative APOBEC3 mutations, versus the original 140). Finally, we cannot assume that intact *env-gp120* sequences come from fully intact HIV genomes. As such, the comparison group for hypermutated sequences in the present study is not the replication competent HIV reservoir, but rather the pool of proviruses with intact *env-gp120* sequences, many of which will have defects elsewhere.

In summary, the current practice of excluding hypermutated proviruses from phylogenies used for hypothesis testing has been a major barrier to understanding the in vivo evolutionary origins and longevity of these sequences. Here, we validated two simple nucleotide alignment modification approaches that, for the first time, allow hypermutated sequences to be correctly incorporated into phylogenies that can be used for molecular dating. Overall, our observations reveal that hypermutated proviruses, like other provirus types, are archived throughout untreated infection and can persist for years on ART. Our observations further suggest that the evolutionary dynamics of hypermutated proviruses may differ from those of other proviral types in some individuals. In addition to enriching our understanding of HIV persistence towards the ultimate goal of HIV cure, the approaches developed here could be extended to between-host phylogenies, and testing of other hypotheses related to within-host evolutionary origins of hypermutated sequences.

## Figures and Tables

**Table 1:** Participant information, HIV sampling and sequencing details

ID^	Estimated date of infection	Duration of uncontrolled infection (years)	No. of pre-ART plasma HIV RNA time points	Distinct pre-ART plasma HIV *env-gp120* sequences	ART initiation date	Years of ART until last proviral sampling	No. of on-ART proviral time points	Distinct on-ART HIV *env-gp120* proviral sequences (Hypermutated N; %)
WIHS-P2	Jan 2003	9	10	227	Jan 2012	6.8	3	75 (22; 28%)
WIHS-P4*	Jul 1995	10.9	9	182	Jun 2006	12.3	4	155 (23; 15%)
WIHS-P1	Dec 1995	12	13	207	Jan 2008	10.3	4	85 (15; 13%)
WIHS-P3	Jul 2002	5.5	9	132	Jan 2008	8.8	3	59 (5; 8%)
WIHS-P5	Mar 2008	1.9	2	45	Feb 2010	8.7	3	74 (22; 30%)
WIHS-P6	Aug 2006	3.9	6	73	Jul 2010	8.3	4	94 (6; 6%)

^ Participants are numbered in the same order as the original manuscript (Shahid et al, 2024). That is, WIHS-P2 in the present study is Participant 2 in (Shahid et al, 2024).

* The MWCCS database indicated that participant 4 initiated ART in 2003, but no reductions in plasma viral load (pVL) were observed until June 2006. For this reason, we considered June 2006 as this participant’s effective ART start date.

**Table 2:** Hypermutated sequence details

ID	Hypermutated proviruses	Aligned HIV *env-gp120* sequence length (bp)	Putative hypermutated nucleotide positions in the alignment^a^	Hypermutated sites identified per sequence Median (range)^b^
WIHS-P2	22	1515	140	43 (20 – 68)
WIHS-P4	23	1541	176	55 (34 – 83)
WIHS-P1	15	1483	141	41 (10 – 75)
WIHS-P3	5	1486	127	40 (36 – 64)
WIHS-P5	22	1501	152	57 (9 – 78)
WIHS-P6	6	1523	122	47 (35 – 75)

a The total number of nucleotide positions that harbored an A at an APOBEC3 target site in at least one hypermutated sequence in the participant’s sequence alignment. These positions were stripped out of the alignment in the HM-Stripped approach.

b Statistics summarizing the overall number of A bases at APOBEC3 target sites in the participant’s hypermutated sequences. These A bases were changed to R or G, respectively, in the HM-Replacedw/R and HM-Replacedw/G approaches.

## Data Availability

The nucleotide sequences reported in this paper are available in GenBank (proviral DNA accession numbers: OR404056 - OR404777, OR404820 - OR404981; HIV RNA accession numbers: OR403057 - OR403738).
